# Editorial: Using the RE-AIM framework and other implementation theories, models, and frameworks to guide the implementation and evaluation of rural health innovations

**DOI:** 10.3389/frhs.2025.1695418

**Published:** 2025-10-14

**Authors:** Heather Schacht Reisinger, Teresa Damush, Erin Balkenende, Edwin Wong, Kanika Arora, Rachael R. Kenney, Laura D. Taylor, Monica Matthieu

**Affiliations:** ^1^ Center for Access and Delivery Research and Evaluation (CADRE), Iowa City VA Health Care System, Iowa City, IA, United States; ^2^ Department of Internal Medicine, Carver College of Medicine, University of Iowa, Iowa City, IA, United States; ^3^ Implementation Science Center, University of Iowa, Iowa City, IA, United States; ^4^ Center for Health Information and Communication (CHIC), VA Indiana Healthcare System, Indianapolis, IN, United States; ^5^ Department of General Internal Medicine and Geriatrics, Indiana University School of Medicine, Indianapolis, IN, United States; ^6^ Regenstrief Institute, Inc., Indianapolis, IN, United States; ^7^ Center of Innovation for Veteran-Centered and Value Driven Care, VA Puget Sound Health Care System, Seattle, WA, United States; ^8^ Department of Health Systems and Population Health, University of Washington, Seattle, WA, United States; ^9^ Department of Health Management and Policy, College of Public Health, University of Iowa, Iowa City, IA, United States; ^10^ Center of Innovation for Veteran Centered and Value Driven Care, VA Eastern Colorado Health Care System, Aurora, CO, United States; ^11^ Veterans Health Administration, VA Central Office, Patient Care Services, Washington, DC, United States; ^12^ U.S. Department of Veterans Affairs Medical Center, Central Arkansas Veterans Healthcare System, HSR&D Center of Innovation: Center for Mental Healthcare & Outcomes Research, Little Rock, AR, United States; ^13^ School of Social Work, Saint Louis University, Saint Louis, MO, United States

**Keywords:** rural health, theories, models, and frameworks, RE-AIM, veterans, evaluation

**Editorial on the Research Topic**
Using the RE-AIM framework and other implementation theories, models, and frameworks to guide the implementation and evaluation of rural health innovations

While many of the challenges facing rural health are well described (1–5) —from divestment (6) and disparities (7) to long travel distances to care (8–10) and workforce shortages (11, 12) —the role implementation science can play in addressing those barriers is less well-addressed (13, 14). Theories, models, and frameworks can guide systematic planning and evaluation of implementation that addresses these barriers (15). They also allow for comparison across innovations and implementation contexts through the examination of shared constructs (13). The goal of this Research Topic and its compilation of articles is to examine challenges of implementation and evaluation in the rural healthcare context, using theories, models, and frameworks to guide the discussion.

In 2006, the United States Congress passed 38 U.S. Code § 7,308 thereby establishing the Department of Veterans Affairs (VA) Office of Rural Health (ORH). The mission of VA ORH is to improve the health and well-being of rural veterans through research, innovation and dissemination of best practices. ORH fulfills this mission with three pillars: (1) to promote system-wide and community care solutions for rural veterans, (2) to reduce rural health care workforce disparities, and (3) to enrich rural veteran health research and innovation. One of the ways VA ORH supports its mission is through the funding of enterprise-wide initiatives (EWIs) that seek to spread evidence-based interventions and best practices to rural veterans across the United States (https://www.ruralhealth.va.gov/providers/enterprise_wide_initiatives.asp). Integrated into the EWI program is an evaluation requirement guided by the planning, evaluation, and implementation framework, RE-AIM (Reach, Effectiveness, Adoption, Implementation, and Maintenance) (16, 17).

ORH's EWI program's use of the RE-AIM framework to structure its evaluation requirements presents a unique opportunity to examine real-world implementation of innovations through a standard lens. In most implementation research, the investigative team decides on the intervention, setting, and population; designs the research questions; determines the implementation strategies; and oversees the conduct and analysis of the methods to reach the results. In the context of ORH's EWIs, evaluation teams, often with considerable health services and implementation science expertise, are paired with VA clinical operational and field-based leads to test the implementation of best practices and innovations. In partnership, the operational, field-based, and evaluation teams decide on the evaluation design and outcome measures and the evaluation team then conducts the agreed upon evaluation. The impetus for this Research Topic was to bring together a variety of EWI evaluations and provide examples of how using the RE-AIM framework may lead to broader lessons learned for large-scale implementation of rural health innovations. The Research Topic was also an opportunity to examine other large-scale evaluation programs that focus on rural health, particularly those that have integrated theories, models, and frameworks to guide their efforts. The result is a compilation of 19 papers at the intersection of rural health and implementation science primarily in the VA context, although it includes large-scale programs in the U.S. community health sector (Melhado et al.; Petermann et al.) and a systematic review focused on rural youth accessing health care across the 54 countries in Africa (Gbaja-Biamila et al.). Importantly, the lessons learned are cross-cutting and provide insight into directions implementation science could take to improve rural health globally.

We compiled Table 1 to assist researchers in quickly determining which articles in the compilation could best inform their work. Overall, 15 of the 19 articles applied RE-AIM as their framework, which is not surprising given the focus of the special issue and the number of publications on EWIs (*n* = 14). One additional publication utilized RE-AIM in the context of the Practical, Robust Implementation and Sustainability Model (PRISM). Two articles applied Consolidated Framework for Implementation Research (CFIR) and one used the Critical Incident Technique as a framework to more fully examine sustainability.

**Table 1:** Characteristics of articles in the research topic compilation.

Author	Article Title	VA?	EWI?	TMF	Barriers and Facilitators?	Method
Belkora	Sustainment of the TeleSleep Program for Rural Veterans	Y	Y	Critical Incident Framework	1	Mixed
Chasco	RE-AIM for Rural Health Innovations: Perceptions of (Mis) Alignment between the RE-AIM Framework and Evaluation Reporting in the Department of Veterans Affairs Enterprise-Wide Initiative Program	Y	Y	RE-AIM	1	Qual
Cornell	Benefits and challenges in the use of RE-AIM for evaluation of a national social work staffing program in the veterans health administration	Y	Y	RE-AIM	1	Quan
Damush	The VA National TeleNeurology Program (NTNP) Implementation: A Mixed-Methods Evaluation Guided by RE-AIM Framework	Y	Y	RE-AIM	1	Mixed
Gbaja-Biamila	Interventions connecting young people living in sub-Saharan Africa to healthcare; A systematic review using the RE-AIM Framework	N	N	RE-AIM	1	Review
Golden	RE-AIM Applied to a Primary Care Workforce Training for Rural Providers and Nurses: The Department of Veterans Affairs’ Rural Women's Health Mini-Residency	Y	Y	RE-AIM	1	Quan
Gould	Implementation of Tele-Geriatric Mental Health Care for Rural Veterans: Factors Influencing Care Model Variations	Y	N	CFIR	1	Qual
Jackson	Diffusion of Excellence: Evaluating a System to Identify, Replicate, and Spread Promising Innovative Practices across the Veterans Health Administration	Y	Y	RE-AIM	1	Mixed
Kenney	Applying RE-AIM to Evaluations of United States Veterans Health Administration Enterprise-Wide Initiatives: Lessons Learned	Y	Y	RE-AIM	1	Qual
Lamkin	Using the RE-AIM Framework to Assess National Teledermatology Expansion	Y	Y	RE-AIM	1	Mixed
Leonard	Implementation of the Mobile Prosthetic and Orthotic Care (MoPOC) Program in the VA; A Qualitative Study of Implementation Challenges and Associated Strategies for Improvement	Y	Y	RE-AIM	1	Qual
Lewis	Rural Barriers and Facilitators of Lung Cancer Screening Program Implementation in the Veterans Health Administration: A Qualitative Study	Y	Y	RE-AIM	1	Qual
Matthieu	Adopting the RE-AIM analytic framework during program implementation: Experiences from the Advance Care Planning via Group Visits national evaluation	Y	Y	RE-AIM	1	Quan
Mattocks	Using RE-AIM to Examine Implementation of a Tele-Nephrology Program for Veterans Living in Rural Areas	Y	Y	RE-AIM	1	Qual
Mattox	Utilizing the RE-AIM Framework for a Multispecialty Veterans Affairs Extension for Community Healthcare Outcomes (VA-ECHO) Program 2018–2022	Y	Y	RE-AIM	1	Quan
Melhado	Utilizing PRISM and RE-AIM to implement and evaluate the Rural Telementoring Training Center (RTTC) for health care workforce development in rural communities	N	N	RE-AIM + PRISM	1	Qual
Mignogna	Expanding Access to Evidence-Based Psychotherapy in VA Settings: Implementation of the Brief Cognitive Behavioral Therapy for Depression Program	Y	N	RE-AIM	0	Quan
Petermann	Assessing the Pre-Implementation Context for Financial Navigation in Rural and Non-Rural Oncology Clinics	N	N	CFIR	1	Mixed
Relyea	Evaluating an Enterprise-Wide Initiative to enhance healthcare coordination for rural women Veterans using the RE-AIM framework	Y	Y	RE-AIM	1	Mixed

VA, U.S. Department of Veterans Affairs; EWI, enterprise wide initiative; TMF, theory, model, framework; Qual, qualitative methods; Quan, quantitative methods; RE-AIM, reach, effectiveness, adoption, implementation, maintenance framework; CFIR, consolidated framework for implementation research; PRISM, practical, robust implementation and sustainability model.

The strength of the majority of publications applying the same framework is that it makes it easier to compare the variation in how a single construct was applied for each individual construct. For example, *reach* is the first construct of the RE-AIM framework, and this focus allows other implementation scientists, rural health researchers, and policy makers to compare multiple issues related to *reach*. For example, are there common barriers or facilitators to reaching an a rural-dwelling population? Are there particular implementation strategies that help programs reach this population? Interestingly, 18 of the 19 articles in the special issue address barriers to implementation in some way and what is unique about this compilation is that all are directly related to the implementation process in rural settings.

In additional, several articles discuss the strengths and weaknesses of applying specific models to evaluating implementation. Chasco et al. and Kenney et al. examine the use of RE-AIM across multiple EWIs and describe the challenges of defining and applying the five RE-AIM domains. Melhado et al. and Leonard et al. elaborate on the use of frameworks (RE-AIM and PRISM + RE-AIM) to iteratively improve implementation of the interventions they focus on in their articles. Overall, most articles reflect Damush et al. review that “the pragmatic application of the RE-AIM framework to guide implementation evaluations is appropriate, comprehensive, and recommended for future applications”.

From a methodological perspective, theories, models, and frameworks are not prescriptive. While a slight majority (*n* = 7) of the articles used qualitative methods to examine the constructs in each program, six relied on a mix of qualitative and quantitative approaches. Quantitative methods were used exclusively in five of the publications and included surveys, validated instruments, and administrative data to answer questions related to the constructs. This compilation of articles provides readers with a rich set of measures across constructs, which will allow researchers to compare these measures and make decisions in their own work about the most effective approaches to use. A few examples of explicit operationalization of RE-AIM constructs are: Cornell et al., Lamkin et al., Matthieu et al., Mattox et al., and Relyea et al.

Our comparison of the articles in this compilation are only an initial review. Other comparisons could be made such as a more in-depth review of barriers to shed light on barriers specific to rural implementation. We look forward to learning of the ways the compilation of these articles together contributes to the scholarship in implementation specific to rural settings. Finally, we anticipate the ability to read these articles as a whole will lead to additional research, evaluation, and policy questions to support improved implementation and outcomes for rural populations. Our goal was to demonstrate the value of applying theories, models, and frameworks in rural health settings and promote the use of implementation science to improve the care and health outcomes of rural patients and community members. We hope this Research Topic and its articles contributes to this conversation.
